# Pushing the limits of sulfur SAD phasing: *de novo* structure solution of the N-terminal domain of the ectodomain of HCV E1

**DOI:** 10.1107/S139900471401339X

**Published:** 2014-07-25

**Authors:** Kamel El Omari, Oleg Iourin, Jan Kadlec, Richard Fearn, David R. Hall, Karl Harlos, Jonathan M. Grimes, David I. Stuart

**Affiliations:** a Division of Structural Biology, Wellcome Trust Centre for Human Genetics, University of Oxford, Oxford OX3 7BN, England; b Diamond Light Source Ltd, Diamond House, Harwell Science and Innovation Campus, Didcot OX11 0DE, England

**Keywords:** sulfur SAD, HCV, envelope glycoprotein E1

## Abstract

Single-wavelength anomalous dispersion of S atoms (S-SAD) is an elegant phasing method to determine crystal structures that does not require heavy-atom incorporation or selenomethionine derivatization. Nevertheless, this technique has been limited by the paucity of the signal at the usual X-ray wavelengths, requiring very accurate measurement of the anomalous differences. Here, the data collection and structure solution of the N-terminal domain of the ectodomain of HCV E1 from crystals that diffracted very weakly is reported. By combining the data from 32 crystals, it was possible to solve the sulfur substructure and calculate initial maps at 7 Å resolution, and after density modication and phase extension using a higher resolution native data set to 3.5 Å resolution model building was achievable.

## Abstract 2

The sulfur SAD phasing method was successfully used to determine the structure of the N-terminal domain of HCV E1 from low-resolution diffracting crystals by combining data from 32 crystals.

## 1. Introduction   

Anomalous dispersion methods are powerful techniques to determine protein structures (Hendrickson, 2013 ▶), especially when it is possible to tune the X-ray energy to points close to an absorption edge for atoms within the crystal to maximize the anomalous (Δ*f*′′) and dispersive (Δ*f*′) differences. Multi-wavelength and single-wavelength anomalous dispersion (MAD and SAD) techniques using selenomethionine (SeMet) are nowadays the workhorse methods for the *ab initio* phasing of macromolecular crystals (Hendrickson *et al.*, 1990 ▶). Despite the success of these methods, some proteins have few or no methionines, or the SeMet-labelled protein may be reluctant to crystallize. In the same manner, selenocysteine-labelled proteins can be expressed in non-auxotrophic *Escherichia coli* strains (Salgado *et al.*, 2011 ▶), but this method is likely to encounter the same problems as SeMet-labelled expression, such as lower protein expression, lower solubility or low selenium incorporation in more difficult targets requiring eukaryotic expression systems. Conventional heavy-atom isomorphous replacement methods tend to rely on trial and error (Joyce *et al.*, 2010 ▶) and usually require testing numerous compounds at different concentrations while keeping the scatterer soluble without damaging the crystals. In contrast, single-wavelength anomalous dispersion of S atoms (S-SAD) does not require the use of selenium-labelled protein or heavy-atom incorporation, as phases can be derived directly from the naturally occurring sulfurs of both cysteines and methionines. Although the S-SAD method was successfully used for the first time more than 30 years ago (Hendrickson & Teeter, 1981 ▶), the number of *de novo* structures determined by this method is still limited (Liu *et al.*, 2012 ▶). The absorption edge of sulfur (∼5 Å) cannot be usefully exploited by conventional synchrotron crystallography beamlines, radiation damage is enhanced at longer wavelengths and absorption becomes severe, so S-SAD is usually carried out at shorter wavelengths (λ = 1.5–2.5 Å). As a direct consequence, the anomalous signal of the sulfur is very weak, so a high signal-to-noise ratio is required for satisfactory measurement of the faint signal. The latter ratio can be improved by increasing the multiplicity; however, poorly diffracting crystals require greater X-ray doses and thus obtaining high-multiplicity data sets is often not possible from a single crystal. In order to overcome this problem, the anomalous differences can be recorded from multiple isomorphous crystals until the desired multiplicity is reached while keeping the radiation damage low (Liu *et al.*, 2012 ▶, 2013 ▶). A second method to enhance the anomalous differences in the face of radiation damage is to use the inverse-beam data-collection strategy (Hendrickson *et al.*, 1989 ▶). The Friedel mates (*h*, *k*, *l*) and (−*h*, −*k*, −*l*) are recorded in small wedges at ϕ and ϕ + 180°, ensuring that Friedel pairs are recorded close in time while minimizing the difference in absorption effects and radiation damage.

S-SAD phasing was applied to determine the structure of the N-terminal domain of the ectodomain of *Hepatitis C virus* envelope glycoprotein E1 (HCV nE1). The HCV envelope glycoproteins E1 and E2 are located on the surface of the virions and are responsible for binding of the virus to the host cells and membrane fusion. Although HCV is a major global health problem, its mechanism of fusion is still not known owing to the lack of structural knowledge of these two glycoproteins. HCV nE1 is composed of 79 residues and contains no methionines, which makes this construct unsuitable for SeMet phasing. Heavy-atom soaking experiments were attempted but failed to show any useful anomalous signal for substructure determination; therefore, efforts were focused on S-SAD methods.

## 2. Methods and results   

### 2.1. Cloning, expression and protein purification   

**Figure 1:**
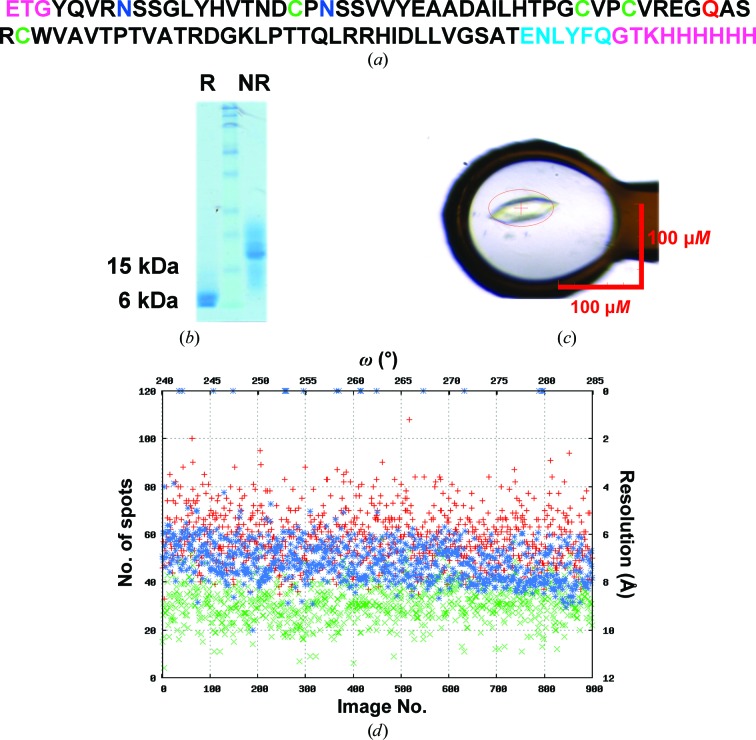
Construct and data-collection details. (*a*) Amino-acid sequence of HCV nE1. Cysteines, glycosylation sites and the N43Q mutation are shown in green, blue and red, respectively. The extra residues resulting from cloning are coloured light blue (TEV cleavage site) and pink. (*b*) 15% reducing (R) and nonreducing (NR) SDS–PAGE gels showing the purity of deglycosylated HCV nE1. (*c*) Typical HCV nE1 crystal. The red ellipse represents the size of the beam. (*d*) *DISTL* plot showing the number of spots and estimated resolution for each image (or ω) in a representative wedge (Zhang *et al.*, 2006 ▶). The number of found spots (red), potential Bragg candidates (green) and the resolution (blue) are depicted as crosses.

DNA coding for the ectodomain of HCV E1 (residues 1–79) was synthesized with a mutation at one of the glycosylation sites (N43Q) and was cloned into the pHLsec vector (Aricescu *et al.*, 2006 ▶). The construct containing a C-terminal His_6_ tag (Fig. 1 ▶ *a*) was transiently expressed in HEK293T cells in the presence of 5 µ*M* kifunensine to limit *N*-glycosylation of the remaining sites (Toronto Research Chemicals, North York, Ontario, Canada). Ni^2+^-affinity purification (FF Chelating Sepharose resin, GE Healthcare) was followed by TEV protease and endoglycosidase F1 treatment before size-exclusion chromatography on a Superdex 75 column (GE Healthcare). The protein was estimated to be greater than 95% pure by SDS–PAGE (Fig. 1 ▶ *b*). 3-(1-Pyridino)-1-propanesulfonate (NDSB 201; Soltec Ventures Inc.) was added to HCV nE1 to a final concentration of 300 m*M* in order to reach concentrations of between 17 and 22 mg ml^−1^.

### 2.2. Crystallization   

A Cartesian Technologies MIC4000 robot was used to set up high-throughput crystallization trials using the sitting-drop vapour-diffusion method at 294 K in 96-well plates (Greiner Bio-One Ltd, Stonehouse, England; Walter *et al.*, 2003 ▶, 2005 ▶). Initial crystal hits for HCV nE1 N43Q were obtained in 15%(*w*/*v*) PEG 1500, 3.6%(*w*/*v*) PEG 4000, 0.05 *M* sodium acetate pH 4.8 (Pi–PEG screen, Jena Bioscience). Crystals of hexagonal morphology appeared after a few days but diffracted extremely weakly and appeared to be twinned. The same condition after some two weeks gave crystals of tetragonal morphology, which were optimized using an additive screen (Hampton Research). Addition of 100 nl of 6–8% 2,5-hexanediol or 1,6-hexanediol to the initial condition improved the size of the crystals to 110 × 30 × 10 µm (Fig. 1 ▶ *c*). Crystals were flash-cooled in liquid nitrogen using 25%(*v*/*v*) ethylene glycol in the reservoir solution as a cryoprotectant.

### 2.3. Data collection   

An initial data set was recorded at 100 K on the I24 beamline at Diamond Light Source (DLS), Didcot, England at a wavelength of 0.9796 Å using a PILATUS 6M detector (DECTRIS) with the crystal-to-detector distance set to 623.5 mm to cover diffraction to 3 Å resolution at the detector edge. A total crystal rotation range of 90° was collected from a single crystal with an exposure time of 0.2 s per 0.1° (100% beam transmission: 10^12^ photons s^−1^ with a beam size of 30 × 30 µm). The space group *P*4_1_2_1_2 (or *P*4_3_2_1_2) and unit-cell parameters *a* = *b* = 105.0, *c* = 204.8 Å, α = β = γ = 90° were obtained by processing the data with *HKL*-2000 (Otwinowski & Minor, 1996 ▶). The data extended to ∼3.5 Å resolution (Table 1 ▶).

HCV nE1 contains 79 residues, two glycosylation sites, four cysteines and no methionines (Fig. 1 ▶ *a*). For a solvent content of 52%, the asymmetric unit would comprise 13 molecules (*V*_M_ of 2.4 Å^3^ Da^−1^), although the very weak diffraction suggested that the solvent content might be higher. From comparison of reducing and nonreducing SDS–PAGE gels (Fig. 1 ▶ *b*), HCV nE1 forms covalent dimers (in agreement with size-exclusion chromatography; data not shown). We did not know whether all of the cysteine residues would be involved in disulfide bonds, but speculated that this was quite likely and recognized that this would enhance the phasing power at very low resolution, where the bonded atoms would scatter coherently, and simplify the determination of the sulfur sub­structure. A calculated Bijvoet ratio of 1.1% (for four free cysteines, or 1.7% for four cysteines involved in disulfide bridges) for the total reflection intensities led us to target an overall signal-to-noise ratio of at least 30 for effective phasing (this guide figure was based on the expectation that the substructure could be determined from the stronger lower resolution reflections). For S-SAD experiments, data sets from 32 randomly orientated crystals were recorded at a wavelength of 1.7712 Å using the inverse-beam method on the I04 beamline at DLS using a PILATUS 6M detector (DECTRIS) with the crystal-to-detector distance set to 560 mm to cover diffraction to 4.5 Å resolution at the detector edge (a helium path was not used). A beam size of 80 × 45 µm was used with a flux of 1.5–2.0 × 10^11^ photons s^−1^. Each crystal was rotated 180° from the initial position every 5° to measure Friedel pairs. On average a total of 90° was collected per crystal in two wedge series (*A* and *B*) of 9 × 5° each with a rotation of 0.05° and an exposure time of 0.05 s per frame. The 64-wedge series (57 600 frames in total) was auto-processed and merged with *xia*2 (Winter *et al.*, 2013 ▶) with good statistics: overall *R*_merge_, completeness and multiplicity of 0.16, 0.99 and 121, respectively. The quality of the merging was reflected in the small number of rejections (0.25%). Data-collection details are shown in Table 1 ▶, which also reports, for comparison purposes, statistics for a typical S-SAD wedge. The rationale for the choice of data-collection parameters is given below.

In order to mitigate absorption effects at longer wavelengths while being able to collect a useful sulfur anomalous signal, the beam wavelength was tuned to 1.77 Å (*f*′′ = 0.7 electrons). It was also crucial to know the lifetime of the crystals when exposed to X-rays. At the selenium edge wavelength at I24, HCV nE1 crystals lasted about 180 s, but to test the behaviour of the crystals at λ = 1.77 Å at I04 we assessed the crystal decay by looking at the number of observed spots per image and finally collected 90 s per crystal (Fig. 1 ▶ *d*). With the aim of maximizing the signal-to-noise ratio, very small rotation angles of 0.05° per image were collected on a PILATUS 6M detector (DECTRIS) operating in shutterless mode (across the 100 images of each 5° wedge) and in order to collect the data sets quickly we used a non-attenuated beam (1.5–2.0 × 10^11^ photons s^−1^) with a very limited exposure time of 0.05 s. A beam size of 80 × 50 µm was used to match the size of the crystals.

Because it was not possible to obtain high multiplicity from a single HCV nE1 crystal, an overall multiplicity of 121 (4.2 in the outer shell) was built up by collecting data sets from 32 crystals. The scaling of all data sets was of excellent quality, with *R*_merge_ and *R*_p.i.m._ values of 0.16 and 0.017, respectively, for the overall data and of 0.35 and 0.24, respectively, for the outer shell. Although the crystal-to-detector distance was set to record reflections to 4.5 Å resolution at the edge, multiple crystals in random orientations permitted full coverage of reciprocal space and allowed the resolution to be extended to 4.2 Å (the corner of the detector) with a CC_1/2_ (Karplus & Diederichs, 2012 ▶) of 0.99 overall and of 0.82 for the highest resolution shell. The anomalous signal extends to 6.7 Å resolution according to *XSCALE* (Kabsch, 2010*a* ▶,*b* ▶) {[|*F*(+) − *F*(−)|/σ] of 1.1 with an anomalous correlation of 31%}, with an overall anomalous multiplicity of 66. Combining multiple crystals for low-resolution phasing has previously been shown to be useful for structure determination in difficult cases (Liu *et al.*, 2013 ▶). An efficient inverse-beam mode method was specifically implemented at the beamline for automatic data collection which allows the recording of accurate Friedel pairs to be prioritized over data completeness. Each crystal was rotated 180° from the starting position every 5° and a total of 90° was collected per crystal in two wedges of 45°. It was essential that the crystals were isomorphous in order to merge them; indeed, merging data from sufficiently non-isomorphous crystals would degrade the anomalous signal. Programs such as *BLEND* (Foadi *et al.*, 2013 ▶) select the optimal clusters of data sets from multiple crystals prior to scaling and merging. In our case, the 32 crystals (64 sweeps) were analysed for isomorphism, and all wedges shared, on pairwise comparison, correlation coefficients of at least 0.92 (0.97 on average) and r.m.s. deviations of 0.26 and 0.45 Å in the *a* and *c* unit-cell parameters, respectively. *BLEND* calculated a linear cell variation of 1.25% between the 64 sweeps (this is the maximum linear change in the diagonals on the three independent cell faces; Foadi *et al.*, 2013 ▶), suggesting that all 64 wedges should be merged in *xia*2 (Winter *et al.*, 2013 ▶) to give the statistics shown in Table 1 ▶.

### 2.4. Structure determination and refinement   

**Figure 2:**
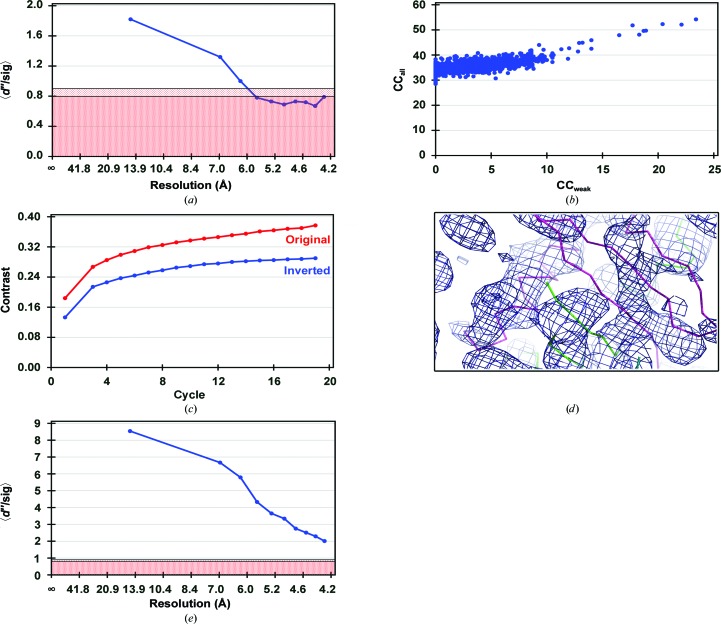
*HKL*2*MAP* profiles. (*a*) *d*′′/sig(*d*′′) as a function of resolution. The graph shows the signal to noise from the anomalous differences. In the red part of the graph the anomalous signal is considered to be nonexistent. (*b*) Profiles of correlation coefficients between observed and calculated Bijvoet differences. (*c*) Contrast between the variance in the electron density in the protein region and in the solvent region for a given phase set as a function of cycle number with phases calculated based on the original (red) or inverted (blue) substructure. (*d*) Initial experimental electron-density maps at 7 Å resolution (original) contoured at at 1σ obtained from *SHELXE*; the final model has been displayed to assess the map quality. (*e*) *d*′′/sig(*d*′′) as a function of resolution as in (*a*) but using calculated anomalous differences from the final refined HCV nE1 model.

The sulfur substructure was determined using the *HKL*2*MAP* graphical interface (Pape & Schneider, 2004 ▶) with *SHELXC*, *SHELXD* and *SHELXE* (Sheldrick, 2010 ▶). *SHELXC* showed a weak anomalous signal extending to about 6.5–7 Å resolution (Fig. 2 ▶ *a*). It was initially difficult to locate any sulfur sites with* SHELXD* as the crystals have an even higher solvent content than expected (six molecules in the asymmetric unit, corresponding to 75% solvent content with a *V*_M_ of 4.9 Å^3^ Da^−1^); thus, the number of sites searched for was initially overestimated. After performing multiple runs (1000 trials per run) with different numbers of heavy-atom sites and resolution cutoffs, a solution could be obtained for 12 S atoms at 7 Å resolution (in the most favourable case the success rate was 0.8%; Fig. 2 ▶ *b*). The main criterion for selecting a probable number of sulfur sites in the asymmetric unit was to select the *SHELXD* runs which gave the highest CC_all_ and CC_weak_ and to judge the number of sites by the occupancies. For six molecules in the asymmetric unit, we expected that the 24 sulfurs might be involved in disulfide bonding, but at such low resolution a disulfide bond would scatter coherently as a single heavy atom (Debreczeni *et al.*, 2003 ▶; Usón *et al.*, 2003 ▶; the transverse coherence length of the X-ray beam is more than four orders of magnitude greater than this bond length). The correctness of the solution was confirmed by *SHELXE*, which showed a separation in the map contrast between the two hands (0.377 *versus* 0.290), implying that the correct space group was *P*4_1_2_1_2 and not *P*4_3_2_1_2 (Fig. 2 ▶ *c*); nevertheless, the initial maps were not readily interpretable (Fig. 2 ▶ *d*).

**Figure 3:**
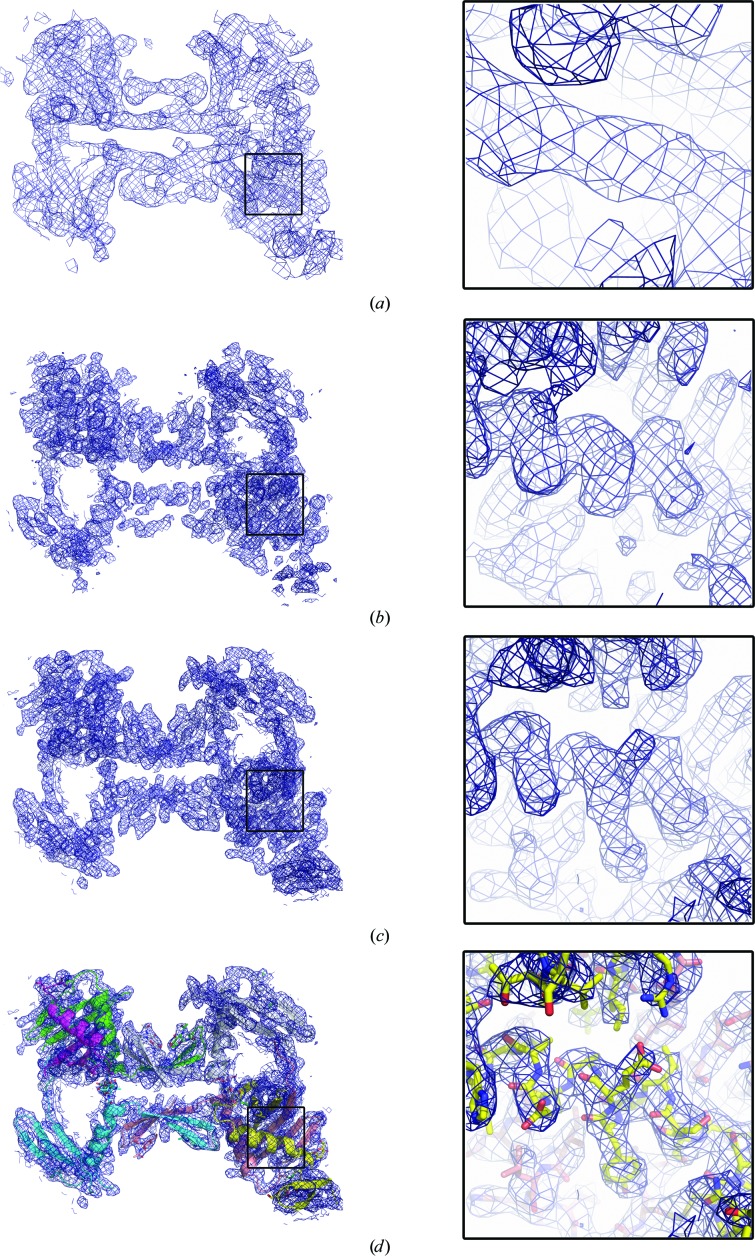
Improvement of electron-density maps. The blue meshes show the electron density contoured at 1σ. (*a*) Electron-density maps at 7 Å resolution after density modification by *phenix.autosol* using a solvent content of 75%. (*b*) Electron-density maps at 3.5 Å resolution after density modification by *phenix.autobuild* using sixfold NCS. (*c*) Final 2|*F*_o_| − |*F*_c_| electron-density maps at 3.5 Å resolution after refinement with *autoBUSTER*. (*d*) Structure of HCV nE1 fitted into the electron-density maps described in (*c*). The six monomers composing the aymmetric unit are coloured differently.

SAD phasing was performed by *phenix.autosol* (Adams *et al.*, 2002 ▶) using the sulfur sites obtained by *HKL*2*MAP* (Pape & Schneider, 2004 ▶). It was essential to cut the resolution to 7 Å and set the solvent content to 0.7 to obtain initial phases (Fig. 3 ▶ *a*) and only then extend to the full resolution (4.2 Å); however, the software was not able to automatically determine the NCS operators, so rebuilding was not feasible. Nonetheless, it was possible to identify density possibly corresponding to α-helices. Six α-helices were located in the map and manually fitted using *Coot* (Emsley & Cowtan, 2004 ▶), keeping the same orientation within each monomer (at this resolution the helix directionality could not be determined); noncrystallographic symmetry (NCS) operators were then calculated using *phenix.find_ncs_operators* (Adams *et al.*, 2002 ▶). These were then input to *phenix.autobuild* (Adams *et al.*, 2002 ▶) with the higher resolution data set (FP and SIGFP), initial maps (phases) and heavy-atom positions (which helped with the NCS determination). Density modification using a solvent content of 75%, sixfold NCS averaging and extension of the resolution to that of the native data set (3.5 Å resolution) resulted in interpretable maps (Fig. 3 ▶ *b*). Secondary structures were clearly visible (Fig. 3 ▶ *b*) and a partial structure could be built using *Coot* (Emsley & Cowtan, 2004 ▶). Refinement using *autoBUSTER* with local structure symmetry and external (S-SAD) phase restraints (Bricogne *et al.*, 2008 ▶), alternating with rebuilding using *Coot*, taking into account cysteine positions (four per monomer, all involved in disulfide bonds) and glycan positions (two per monomer), led to a reliable structure and excellent quality electron-density maps. Refinement statistics are given in Table 1 ▶. As expected, the quality of the maps benefited from the 75% solvent content (Watanabe *et al.*, 2005 ▶) and sixfold NCS (Figs. 3 ▶ *c* and 3 ▶ *d*). The structure will be described elsewhere (manuscript submitted) and the coordinates and structure factors have been deposited in the Protein Data Bank as entry 4uoi.

**Figure 4:**
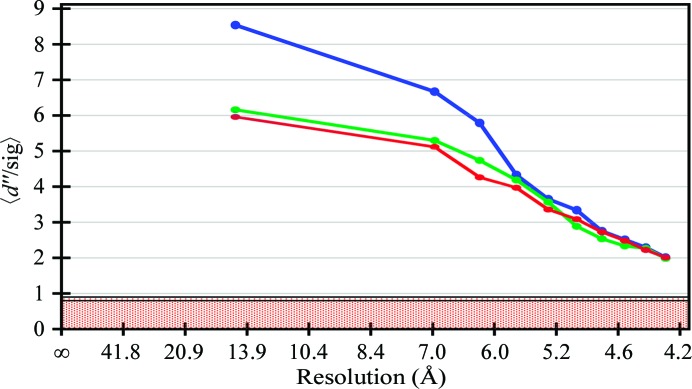
Calculated anomalous differences. Calculated *d*′′/sig(*d*′′) from refined structures as a function of resolution. The graph shows the signal to noise from the anomalous differences. In the red part of the graph the anomalous signal is considered to be nonexistent. The *d*′′/sig(*d*′′) calculated from the final structure, from a structure with cysteine side chains flipped by 180° and from a structure with S atoms from disulfide bonds moved 10 Å away from each other are coloured blue, green and red, respectively.

From the refined structure, we calculated theoretical anomalous differences using *phenix.fmodel* (Adams *et al.*, 2002 ▶) in order to plot the calculated anomalous signal against resolution. The structure factors were also calculated from structures in which the disulfide bonds were disrupted by rotating each side chain by 180° or by placing S atoms 10 Å away from each other (Fig. 4 ▶). This shows the expected marked increase in anomalous signal at low resolution (below 5.5 Å) when the sulfurs are involved in disulfide bonding, reflecting the coherent diffraction of two sulfurs. At higher resolution this coherence is lost.

## 3. Conclusions   

Recent developments in synchrotron instrumentation and crystallographic software have helped to improve the sulfur SAD phasing method, which is in principle the best technique for structure solution as most native crystals can be directly used for phasing. Practically, the approach is limited by a number of different factors. The work reported here shows that useful phasing can be obtained without the need for high-resolution diffraction, or indeed strongly diffracting crystals, if careful data collection is carried out in order to obtain a highly redundant data set from mutiple crystals; indeed, the useful anomalous signal of HCV nE1 crystals did not extend to better than 6.5 Å resolution. The nature of the crystals is also very important; in our case we benefitted from isomorphous crystals, facilitating the scaling and merging of the data, whilst a high solvent content and NCS improved the quality of the early maps. We expect that future hardware and software development will increase the success rate of sulfur phasing and increasingly render it the method of choice for *ab initio* phasing.

## Supplementary Material

PDB reference: N-terminal domain of the ectodomain of HCV E1, 4uoi

## Figures and Tables

**Table 1:** Data-collection statistics Values in parentheses are for the highest resolution shell.

	S-SAD	S-SAD	Native
Data collection			
Beamline	I04, DLS	I04, DLS	I24, DLS
Space group	*P*4_1_2_1_2	*P*4_1_2_1_2	*P*4_1_2_1_2
Unit-cell parameters (, )	*a* = *b* = 105.2, *c* = 204.4, = = = 90	*a* = *b* = 105.5, *c* = 204.8, = = = 90	*a* = *b* = 105.0, *c* = 204.7, = = = 90
No. of crystals	1 [1-wedge series]	32 [64-wedge series]	1
Wavelength ()	1.7712	1.7712	0.9686
Resolution ()	42.74.5 (4.644.52)	60.34.2 (4.324.21)	503.5 (3.633.50)
No. of unique reflections	10723 (625)	15823 (1033)	15137 (1471)
Completeness (%)	92.8 (82.2)	99.4 (96.3)	99.5 (99.5)
Multiplicity	3.1 (2.4)	121.5 (4.2)	6.2 (6.2)
*I*/(*I*)	6.8 (2.5)	33.3 (3.6)	17.6 (2.2)
*R*_merge_ † (%)	9.8 (24.1)	16.0 (35.2)	13.5 (81.0)
*R*_p.i.m._ (%)	7.0 (20.9)	1.7 (24.0)	5.5 (35.2)
CC_1/2_, highest resolution shell	0.90	0.82	0.66
Refinement			
Resolution ()			31.33.5
*R*_work_/*R*_free_ (%)			21.6/23.7
R.m.s.d., bond lengths ()			0.008
R.m.s.d., angles ()			1.13
Mean *B* factor (^2^)			88.4
Wilson *B* factor (^2^)			118.2
Ramachandran plot (%)			
Favoured			97.4
Allowed			100
Outliers			0

† *R*_merge_ = 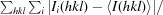 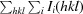, where *I*_i_(*hkl*) is the *i*th measurement of reflection *hkl* and *I*(*hkl*) is the weighted average of all measured reflections.
